# Effect of aerobic exercise training on EEG: event-related potential and neuropsychological functions in depressed elderly with mild cognitive impairment

**DOI:** 10.1590/1980-5764-DN-2022-0082

**Published:** 2023-07-17

**Authors:** Zainab Khan, Ashi Saif, Neera Chaudhry, Adila Parveen

**Affiliations:** 1 Jamia Millia Islamia Central University, Centre for Physiotherapy and Rehabilitation Sciences, New Delhi, India.; 2 Vardhman Mahavir College, Safdarjung Hospital, Department of Neurology, Delhi, India.

**Keywords:** Exercise, Depression, Cognitive Dysfunction, Evoked Potentials, Aged, Exercício Físico, Depressão, Disfunção Cognitiva, Potenciais Evocados, Idoso

## Abstract

Increased depressive severity has been linked to neurocognitive impairment. Aerobic exercise (AE) is an efficient technique for improving cognitive function. However, studies indicating the importance of AE to neurophysiological and neuropsychological functions in the depressed elderly using event-related potentials (ERPs) are scarce.

**Objectives:**

This study aimed to identify the potential benefits of AE on neurophysiological and neuropsychological functions.

**Methods:**

A total of 30 depressed older adults (AE group: n=15; control group (CG): n=14) were recruited based on the inclusion and exclusion criteria. The AE group was subjected to an 8-week-period AE program (3 times/week for 30 min per session) at moderate intensity, determined using heart rate maximum (HRmax). The training intensity was set at 50% HRmax and increased by 5% in subsequent weeks. Pre- and post-training measures for neurophysiological function were tested using ERP-P300 (amplitude-μV and latency-ms) and also for neuropsychological functions using the trail making test (TMT), mini mental status examination (MMSE), and everyday cognition questionnaire (ECog).

**Results:**

In the experimental group, statistically significant improvements were observed when analyzed for all 3 (group-by-time interaction effect, main effect of time, and main effect of group), in both neurophysiological functions (*p<0.001) and neuropsychological functions (*p<0.001), except for ECog scores, where the results were insignificant for the main effect of a group. Correlation analysis demonstrated no association between neurophysiological and neuropsychological functions (*p>0.05).

**Conclusion:**

Findings showed that 8 weeks of AE training may be a promising approach to improve cognitive functions in depressed older adults. However, considering relatively small number of patients, the question arises for effectiveness in other populations.

## RESUMO.

O aumento da gravidade de quadros de depressão tem sido associado a comprometimento neurocognitivo. O exercício aeróbico (EA) é uma técnica eficiente para melhorar a função cognitiva. No entanto, estudos sugerindo a importância de EA para funções neurofisiológicas e neuropsicológicas usando potenciais relacionados a eventos (PRE) em idosos deprimidos são escassos.

**Objetivo:**

Identificar os potenciais benefícios do EA nas funções neurofisiológicas e neuropsicológicas.

**Métodos:**

Vinte e nove idosos deprimidos (grupo EA: n=15; grupo controle: GC: n=14) foram recrutados segundo critérios de inclusão e exclusão. O grupo EA foi submetido a um programa de EA de oito semanas (3 vezes/semana em sessões de 30 minutos cada) em intensidade moderada, determinada pela frequência cardíaca máxima (FCmáx). A intensidade do treinamento foi fixada em 50% da FCmax e aumentada em 5% nas semanas subsequentes. As medidas pré- e pós-treinamento para a função neurofisiológica foram testadas pelo ERP-P300 (amplitude-μV e latência-ms) e também para as funções neuropsicológicas usando o *Trail Making Test* (TMT), o *Mini Mental Status Examination* (MMSE) e o *Everyday Cognition Questionnaire* (ECog).

**Resultados:**

No grupo experimental, melhorias estatisticamente significativas foram observadas quando analisadas para todos os 3 (efeito de interação grupo por tempo, efeito principal do tempo e efeito principal do grupo), em ambas as funções neurofisiológicas (*p<0,001) e funções neuropsicológicas (*p<0,001), exceto para escores ECog, onde os resultados foram insignificantes para o efeito principal de um grupo. A análise de correlação não demonstrou nenhuma associação entre funções neurofisiológicas e neuropsicológicas (*p>0,05).

**Conclusão:**

Os achados mostraram que 8 semanas de treinamento de EA podem ser uma abordagem promissora para melhorar as funções cognitivas em idosos deprimidos. No entanto, considerando um número relativamente pequeno de pacientes, surge a questão da eficácia em outra população.

## INTRODUCTION

Depression accounts for the greatest burden, with approximately one-third of the elderly population suffering from depression^1^. Increased depression severity has been linked to neurocognitive impairments such as episodic memory and executive function, causing difficulties in planning strategies and mental flexibility, which, in turn, leads to impaired motivational and decision-making functions^2^.

Event-related potential (ERP) — P300 is considered an electrophysiological correlate of cognition^3^. It represents mental processes including attention, executive functions, and short-term memory. Two important P300 constituents that have been examined in majority of studies are latency, which has been linked to the speed of information processing, and amplitude, which is proportional to the number of attention resources engaged during task execution; both of these have been investigated in research^3^. Psychophysiological research has examined the neurocognitive deficits and neural activity in patients with depressive disorders using ERPs, particularly P300^4^. Furthermore, research has shown that P300 has consistent psychometric features in both normative and clinically depressed populations, making it an appropriate neural test for examining individual differences in depressed neurocognitive functioning^5^. Previous research has linked depression to lower P300 amplitudes and longer latencies in response to auditory and visual oddball stimuli^6^. However, these findings have not been replicated across the literature, with studies failing to discover P300 amplitude reductions in currently depressed participants when elicited from auditory oddball tasks and when compared to healthy controls^7^.

It seemed reasonable to predict that physical activity (PA) might have an effect on the P300 component for two main reasons. First, as it is believed to reflect the brain activity that underlies the fundamental components of cognition, the P300 is regarded as a useful instrument for the measurement of cognitive function^8^. Second, it is known that subject-to-subject variability in P300 is influenced by background EEG activity variability in the context of the relationship between background EEG activity and ERPs. In fact, individual variations in EEG power and frequency can affect the amplitude and latency of the ERP components^9^. Physical activity (PA) has been shown to have a favorable impact on the P300 component, as was predicted^3^. Aerobic exercise (AE) is a type of physical activity (PA) that is structured to achieve specific fitness goals^10^. AE has been associated both with the betterment of the psychological health^11^ and improvements in cognitive performance both in older adults^12,13^ and young adults^14^. However, there is still lack of studies investigating the effects of physical activity (PA) on ERP-P300 in depressed elderly with cognitive deficits.

Looking at the gaps in the literature, the primary aim of the present study was to evaluate the potential benefits of an aerobic exercise (AE) training program on neurophysiological functions and neuropsychological functions in depressed elderly people with mild cognitive impairment (MCI). The secondary aim was to check whether there is a correlation between neurophysiological and neuropsychological functions. We hypothesized that there will be a significant association between neurophysiological and neuropsychological correlates and that both functions will improve following an AE training program in depressed older adults with MCI.

## METHODS

### Sample size

The number of subjects was determined by the G. Power 3.1.9.2 software using data from the previous study in which changes in ERP-P300 amplitude were observed following PA training^12^. Notably, 28 subjects (14 in each group) were shown to be necessary based on an effect size of 0.37, alpha level of 0.05, and power (1-beta) of 0.80. Total sample size was 30 patients (15 per group), considering for 10% of dropouts.

Screening of subjects for depression and MCI was done using the patient health questionnaire-9 (PHQ-9)^15^ and Montreal Cognitive Assessment (MoCA) scale,^16^ respectively. Subjects were assessed for physical function and balance status using the Berg balance scale (BBS)^17^ in order to ensure their active participation in AE training.

### Participants

Unmedicated depressed older adults having MCI diagnosed by trained neurologist, both male and female, were recruited from the health center of our university. Subjects were included based on the following inclusion criteria: age ≥60 years; DSM-IV criteria for unipolar major depression based on the structured clinical interview for DSM disorders — non-patient version (SCID-I/NP)^18^; subjects were in depressive episode at the time of their recruitment with PHQ scores ≥10^15^; subjects having MCI with MoCA scores between 19 and 25^16^; subjects who were not engaged in any form of physical training for the last 6 months; medically healthy subjects or, if having any chronic medical conditions, these conditions need to be stable; subjects with normal or corrected to normal vision and hearing; and subjects having general understanding of English language and ability to provide informed consent. Exclusion criteria were as follows: medical and febrile illness; medications that could affect cytokine concentrations; immunizations within 4 weeks; any psychotropic medication use within previous 6 weeks; meeting DSM-IV criteria for psychotic, bipolar, or post-traumatic stress disorder; and subjects with a diagnosis of any other psychological disorders, substance abuse, and attempts at suicide within the last 12 months.

**Figure 1:**
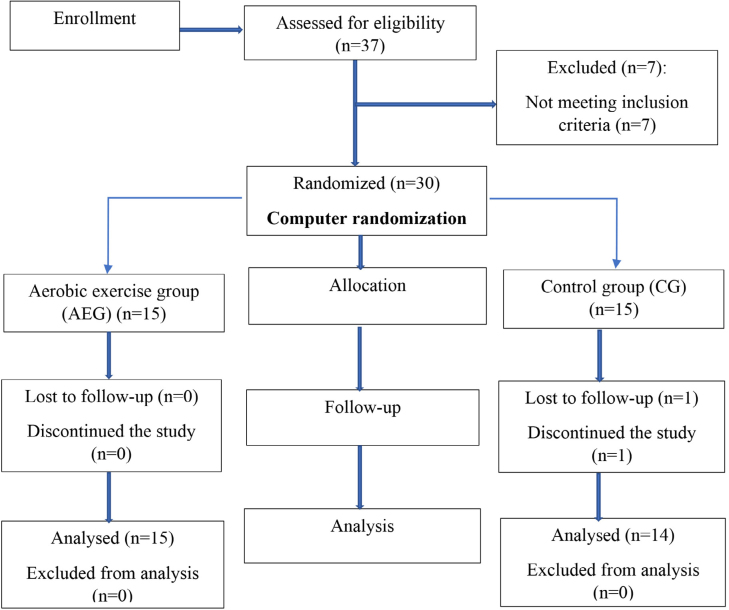
Flowchart showing the study design.

A total of 37 individuals were screened for eligibility, and a total of 30 individuals were included in the study based on the eligibility criteria. Allocation of participants was done by computer randomization, and participants were blinded and allocated to either of the two groups (Figure 1). Demographic details of the participants are shown in Table 1.

**Table 1:** Comparison of anthropometric data between aerobic exercise group and control group using independent sample *t*-test.

Outcome measures	CG (n=14) Mean±SD	AEG (n=15) Mean±SD	p-value	95%CI of difference	
				Lower	Upper
Age (year)	61.90±2.16	61.45±1.43	0.243	-1.18	2.09
Height (cm)	162.29±6.71	163.22±9.01	0.583	-8.01	6.13
Weight (kg)	72.73±6.45	73.93±9.76	0.29	-9.05	5.22
BMI (kg/m^2^)	27.77±4.18	28.07±3.96	0.850	-3.25	3.75
P300 Lat-pre (ms)	305.63±15.37	306.28±16.41	0.683	-14.78	13.50
P300 Amp-pre (μV)	3.94±0.50	3.96±0.48	0.845	-0.46	0.42
TMTA-pre	70.54±3.7	70.63±3.6	0.85	-3.37	3.18
TMTB-pre	174.90±6.60	175.72±6.78	0.82	-6.77	5.14
MMSE-pre	23.45±1.29	22.81±0.75	0.082	-0.30	1.57
ECog-pre	112.63±7.52	113.18±7.20	0.80	-7.10	6.01

Abbreviations: CG: control group; AEG: aerobic exercise group; SD: standard deviation; CI: confidence interval; BMI: body mass index; P300 Lat-pre: P300 latency waves before training; P300 Amp-pre: P300 amplitude waves before training; TMTA-pre: trail making test part A before training; TMTB-pre: trail making test part B before training; MMSE-pre: mini mental state examination before training; ECog-pre: everyday cognition before training. Notes: Data are presented as mean and SD (Mean±SD); significant difference is p<0.05.

### Study procedures

This study was approved by the Institutional Ethical Committee (IEC), Jamia Millia Islamia (Central University), and this clinical trial has been registered prospectively on the Clinical Trial Registry India (CTRI, CTRI/2021/09/036539). The study was communicated to participants during the first contact, and a preliminary diagnostic interview and evaluation of admission requirements were undertaken, following which written informed consent was obtained from participants. Research procedures in this study were performed in conformity with the Declaration of Helsinki, 1964, and its updates. Eligible, consenting subjects then underwent a SCID-I/NP interview to estimate the DSM-IV diagnosis, followed by a comprehensive mental clinical interview and physical examination with the help of a clinical neurologist. The observer-rated PHQ was administered to the depressed subjects to verify the degree of depressive symptom severity. Then, the assessment for the MCI symptoms was done using MoCA in subjects with confirmed depression. Subsequently, outcomes measures involving neurophysiological functions (using ERP-P300) and neuropsychological functions (using trail making test (TMT), mini mental status examination (MMSE), everyday cognition (ECog)) were administered at baseline and post-intervention of the study for each participant. All of the examinations were given in a consistent manner, and the order of the tests was the same for everyone.

## Outcome measures

### Neurophysiological measures

#### ERP-P300 (amplitude and latency)

Pre- and post-training measures for cognitive functions were tested by ERP-P300, including both amplitude (μV) and latency (ms) during wakefulness for 10 min at the same time of the day for all participants to avoid circadian variations on P300 wave. The room temperature was maintained at 26±2°C. Instructions were given to the participants to refrain from consuming caffeinated products and excessive AE for the past 24 h. For the ERP-P300 recording, the participants were made to sit comfortably. The electrodes placement and recording procedure were followed as described in previous studies^19^.

### Neuropsychological measures

#### Cognitive processing speed

TMT-A is used to measure the visual motor-processing speed. On TMT-A, subjects have to connect numbers from 1 to 25, which are randomly spread over a sheet of paper, in ascending numerical order^20^.

#### Executive function

TMT-B is used to measure the executive function. On TMT-B, participants were asked to connect randomly spread numbers (from 1 to 13) and letters (from A to L) in alternating numeric and alphabetical order (1-A-2-B-3-C-…-13-L)^20^.

#### Global cognition

MMSE (for neurocognitive assessment) is composed of 30 items with reference to seven cognitive areas. The total score is between a minimum of 0 and a maximum of 30; a score <19 indicates severe impairment, a score between 19 and 25 indicates MCI, a score of 25 is considered borderline, and a score ≥26 indicates cognitive normality^21^.

#### Everyday cognition

ECog questionnaire measures cognitively relevant functional abilities across six domains with global scoring and contains a total of 39 items. Higher scores indicate greater functional impairment^22^.

### Study intervention

Following baseline measurements, subjects (n=15) assigned to AEG who had not engaged in any form of physical training for the last 6 months undergoes supervised 8-week AE training program (3 times/week, 30 min/session)^13^. Heart rate maximum (HRmax) obtained during the baseline assessment was used to determine the intensity of treadmill training for each participant. Training intensity was initially set at 50% HRmax (5 beats/min), then increased by 5% every week to 65% HRmax (5 beats/min), and then maintained at 65% HRmax from week 4 to week 8 of intervention (i.e., 50% HRmax at first week, 55% HRmax at second week, 60% HRmax at third week, 65% HRmax from weeks 4 to 8). Polar RS400 heart rate monitor was used to track heart rate throughout the training sessions. Each training session began with a 5-min warm-up stretching session and ended with a 5-min cool-down stretching session^23^. The whole AE program was done under the supervision of a physician. All 15 subjects (no dropouts) completed the whole exercise program. No supervised intervention was offered to subjects in the control group (n=14, as there was one dropout).

### Data analysis

Data were analyzed using SPSS version 28.0. Normality distribution of all outcome measures was verified using the Shapiro-Wilk test, skewness, and histogram. Outcome variables that showed a non-normal distribution were analyzed using a non-parametric test or log-transformed. Using an independent sample t-test, baseline measurements and demographic characteristics were examined and found to be comparable between the two groups. All variables were assessed using a 2×2 mixed model ANOVA [group and time effect (pre and post)] for each participant in each group. All comparisons were considered significant at p≤0.05, and the confidence interval was set at 95%. The correlation between neurophysiological and neuropsychological functions was computed using Pearson's correlation analysis.

## RESULTS

All demographic characteristics, including age, weight, height, and BMI, were found to be comparable at the baseline, assessed by independent *t*-test (Table 1). While there was no significant difference between all the outcome variables values in the CG, there was a significant improvement in all the outcome variables values, except ECog scores, when analyzed for main effect of a group in the AEG following 8 weeks of AE training (Table 2).

**Table 2:** Comparison of pre- and post-outcome variables between the groups along-with summary of 2×2 mixed model ANOVA.

Variable	CG (n=14), Mean±SD	AEG (n=15), Mean±SD	Source	f-value	p-value	(η^2^)
P300 Lat-pre (ms)	305.63±15.37	306.28±16.41				
P300 Lat-post (ms)	306.54±14.44	272.95±14.83	T	84.55	<0.001	0.809
			G	6.89	0.016	0.256
			T×G	94.29	<0.001	0.825
P300 Amp-pre (μV)	3.94±0.50	3.96±0.48				
P300Amp-post (μV)	3.79 ±0.45	6.43±0.85	T	53.62	<0.001	0.728
			G	44.71	<0.001	0.691
			T×G	67.75	<0.001	0.772
TMTA-pre	70.54±3.7	70.63±3.6				
TMTA-post	70.63±3.64	55.18±3.51	T	206.99	<0.001	0.912
			G	24.00	<0.001	0.546
			T×G	212.27	<0.001	0.914
TMTB-pre	174.90±6.60	175.72±6.78				
TMTB-post	175.72±6.78	141.09±3.53	T	647.86	<0.001	0.970
			G	45	<0.001	0.69
			T×G	712.07	<0.001	0.973
MMSE-pre	23.45±1.29	22.81±0.75				
MMSE-post	23.18±0.98	26.09±0.70	T	155.57	<0.001	0.88
			G	8.42	0.009	0.29
			T×G	217.28	<0.001	0.916
ECog-pre	112.63±7.52	113.18±7.20				
ECog-post	111.81±6.25	100.45±7.4	T	389.49	<0.001	0.95
			G	3.20	0.088	0.138
			T×G	301.97	<0.001	0.93

Abbreviations: CG: control group; AEG: aerobic exercise group; SD: standard deviation; η^2^: partial eta squared; P300 Lat-pre: P300 latency waves before training; P300 Lat-post: P300 latency waves after training; P300 Amp-pre: P300 amplitude waves before training; P300 Amp-post: P300 amplitude waves after training; TMTA-pre: trail making test part A before training; TMTA-post: trail making test part A after training; TMTB-pre: trail making test part B before training; TMTB-post: trail making test part B after training; MMSE-pre: mini mental state examination before training; MMSE-post: mini mental state examination after training; ECog-pre: everyday cognition before training; ECog-post: everyday cognition after training; T: time; G: group; TxG: time×group. Notes: Data are presented as mean and SD (M±SD); significant difference is p<0.05.

### P300 latency

**Figure 2:**
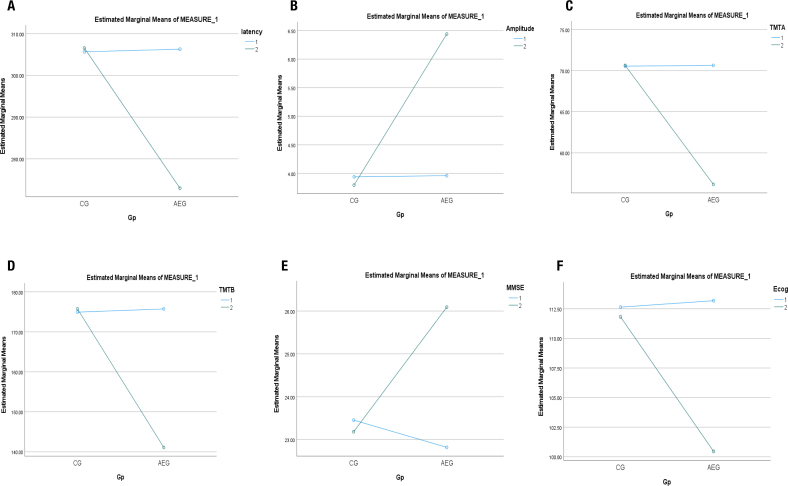
**(A)** Following 8 weeks of aerobic-exercise training, the results indicate an improvement in cognitive function as there is decrease in trend in P300-latency (latency). **(B)** Following 8 weeks of aerobic-exercise training, the results indicate an improvement in cognitive function as there is an increase in trend in P300-amplitude (amplitude). **(C)** Following 8 weeks of aerobic-exercise training, the results indicate an improvement in cognitive function as there is decrease in trend in trail making test part-A (TMTA). **(D)** Following 8 weeks of aerobic-exercise training, the results indicate an improvement in cognitive function as there is a decrease in trend in trail making test part-B (TMTB). **(E)** Following 8 weeks of aerobic-exercise training, the results indicate an improvement in cognitive function as there is increase in trend in mini mental status examination (MMSE) score. **(F)** Following 8 weeks of aerobic-exercise training, the results indicate an improvement in cognitive function as there is decrease in trend in everyday cognition questionnaire (ECog) scores.

Significant improvements were observed between the groups [F(1,27.00)=6.89; p=0.016 (Table 2)], as clinically there was a reduction in latencies of the P300 wave in an AEG compared to a CG following 8 weeks of AE training (Figure 2A). Further significant improvements were observed in the main effect of time [F(1,27.00)=84.55; p<0.001] and in the interaction effect (time x group) [F(1,27.00)=94.29; p<0.001].

### P300 amplitude

Significant improvements were observed between the groups [F(1,27.00)=44.71; p<0.001 (Table 2)], as clinically there was an increment in wave P300-amplitude in the AE group compared to the CG group following 8 weeks of AE training (Figure 2B). Further significant improvements were observed in the main effect of time [F(1,27.00)=53.62; p<0.001] and in the interaction effect (time x group) [F(1,27.00)=67.75; p<0.001].

### Cognitive processing speed

Significant improvements were observed between the groups [F(1,27.00)=24.00; p≤0.001 (Table 2)], as clinically there was a reduction in time taken to complete the TMTA-test by the subjects in an AEG compared to the CG following 8 weeks of AE training (Figure 2C). Further significant improvements were observed in the main effect of time [F(1,27.00)=206.99; p<0.001] and in the interaction effect (time×group) [F(1,27.00)=212.27; p<0.001].

### Executive function

Significant improvements were observed between the groups [F(1,27.00)=45.00; p≤0.001) (Table 2)], as clinically there was a reduction in time taken to complete the TMTB test by the subjects in an AEG compared to the CG following 8 weeks of AE training (Figure 2D). Further significant improvements were observed in the main effect of time [F(1,27.00)=647.86; p<0.001] and in the interaction effect (time×group) [F(1,27.00)=712.07; p<0.001].

### Global cognition

Data showed significant improvements between the groups [F(1,27.00)=8.42; p=0.009 (Table 2)], as clinically there was an increment in MMSE scores in an AEG compared to the CG following 8 weeks of AE training (Figure 2E). Further significant improvements were observed in the main effect of time [F(1,27.00)=155.57; p<0.001] and in the interaction effect (time×group) [F(1,27.00)=217.28; p<0.001].

### Everyday cognition

Data showed no significant main effect between the groups [F(1,27.00)=3.20; p=0.08) (Table 2)], as clinically there was no significant reduction in ECog scores in an AEG compared to a CG following 8 weeks of AE training (Figure 2F). However, significant improvements were observed in the main effect of time [F(1,27.00)=384.49; p<0.001)] and in the interaction effect (time×group) [F(1,27.00)=301.97; p<0.001)].

### Correlations between neuropsychological and neurophysiological functions

**Figure 3:**
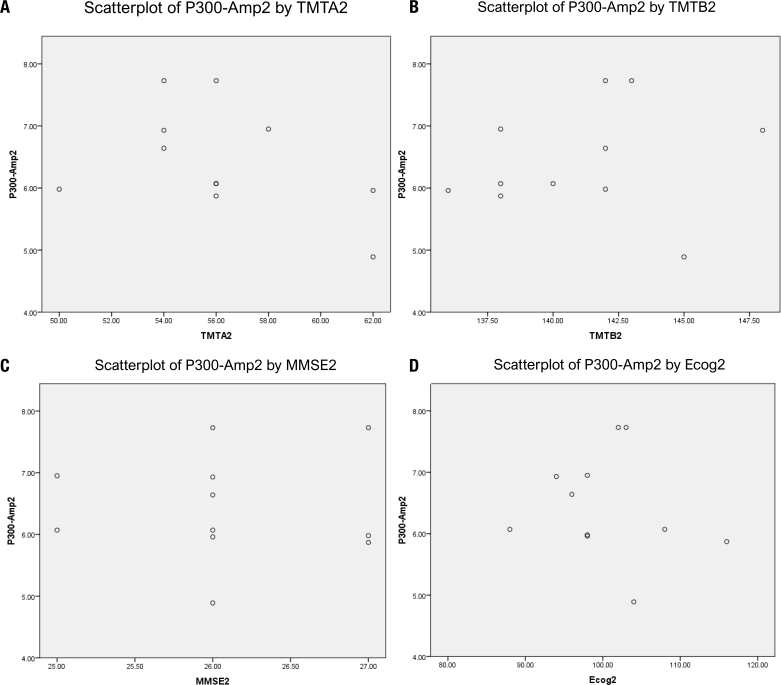
**(A)** A scatterplot representing the result of Pearson correlation coefficients showing no association between post-P300-amplitude (P300-Amp2) and post-trail making test part-A (TMTA2) (r=-0.411, p=0.209). **(B)** A scatterplot representing the result of Pearson correlation coefficients showing no association between post-P300-amplitude (P300-Amp2) and post-trail making test part-B (TMTB2) (r=0.162, p=0.634). **(C)** A scatterplot representing the result of Pearson correlation coefficients showing no association between post-P300-amplitude (P300-Amp2) and post-mini mental status examination score (MMSE2) (r=0.020, p=0.953). **(D)** A scatterplot representing the result of Pearson correlation coefficients showing no association between post-P300-amplitude (P300-Amp2) and post-everyday cognition questionnaire score (ECog2) (r=-0.161, p=0.63).

To address this, we correlated the post-intervention neurophysiological correlates values (post-p300 amplitude — P300 Amp2 and post-p300 latency — P300 Lat 2) with the post-intervention neuropsychological correlates values (post-cognitive processing speed, measured by TMTA=TMTA2; post-executive function, measured by TMTB=TMTB2; post-global cognition, measured by MMSE = MMSE2; and post everyday cognition, measured by ECog=ECog2) in an AE training group using Pearson's correlation. The relationships between P300 Amp2 and TMTA2 (r=-0.411, p=0.209; Figure 3A), between P300 Amp2 and TMTB2 (r=0.162; p=0.634; Figure 3B), between P300 Amp2 and MMSE2 (r=0.020, p=0.953; Figure 3C), and between P300 Amp2 and ECog2 (r=-0.161, p=0.63; Figure 3D) were computed, but they did not show any association.

**Figure 4:**
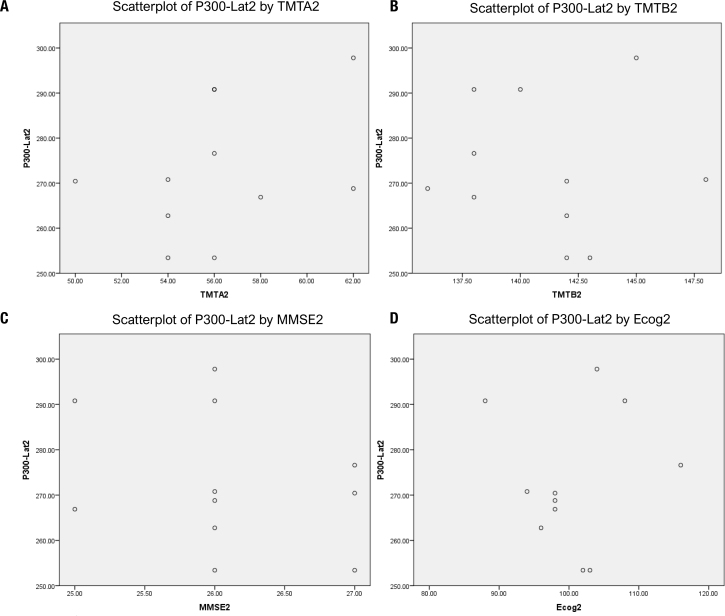
**(A)** A scatterplot representing the result of Pearson correlation coefficients showing no association between post-P300-latency (P300-Lat 2) and post-trail making test part-A (TMTA2) (r=0.366, p=0.268). **(B)** A scatterplot representing the result of Pearson correlation coefficients showing no association between post-P300-latency (P300-Lat 2) and post-trail making test part-B (TMTB2) (r=-0.058, p=0.865). **(C)** A scatterplot representing the result of Pearson correlation coefficients showing no association between post-P300-latency (P300-Lat 2) and post-mini mental status examination score (MMSE2) (r=-0.290, p=0.386). **(D)** A scatterplot representing the result of Pearson correlation coefficients showing no association between post-P300 latency (P300-Lat 2) and post-everyday cognition questionnaire score (ECog2) (r=0.061, p=0.857).

Similarly, a correlation analysis was also done for P300 Lat 2 and TMTA2 (r=0.366, p=0.268; Figure 4A), for P300 Lat 2 and TMTB2 (r=-0.058, p=0.865; Figure 4B), for P300 Lat 2 and MMSE2 (r=-0.290, p=0.386; Figure 4C), and for P300 Lat 2 and ECog2 (r=0.061, p=0.857; Figure 4D), but no association was found in any of the analysis. Therefore, the results point out that neurophysiological functions measurements cannot be replaced by neuropsychological functions tests or vice versa; rather, higher brain functions should be evaluated by both methods.

## DISCUSSION

This study was undertaken to find out the effect of 8 weeks of AE training on cognitive functions in depressed older adults with MCI. Our results indicate that cognitive functions improve as a result of 8 weeks of AE training; this is consistent with many other studies that have found improvements in both P300 amplitude and P300 latencies^24^, whereas a few studies assessed the effects only on P300 latency^25^ after PA training. Furthermore, a systematic review had been carried out, which demonstrated that PA has favorable impact on cortical activities associated with cognitive functions, indicated by P300, in older adults^26^.

Various mechanisms have been put forward for explaining the improvement in P300 waves characteristics with long periods of AE training or exercise. P300 is produced by several cortical generators in the multimodal association cortex around the temporal-parietal junction, which is regulated by the hippocampus formation, according to neurophysiological evidence^3^. The latency and amplitude of P300 are affected by physiological arousal^3^, and a general explanation of P300 physiology based on cholinergic-catecholaminergic interactions within the catecholamine arousal system has been proposed^27^. Furthermore, there is an evidence that AE produces its exercise-induced activation (EIA) effects on auditory P300 by increasing arousal. This evident ability of exercise-engendered physiological arousal to affect P300 latency and P300 amplitude offers a unified mechanism for EIA. As a result, AE appeared to boost the speed of the brain processes underlying the components of cognition such as attention, assessment, and classification, resulting in a decrease in P300 latency overall. Importantly, it is known that exercise increases P300 amplitude^28^ and, in support of this view, this study found that auditory P300 amplitude increased after AE training. According to some P300 theories^29^, the amplitude reflects attentional allocation and context updating of working memory resources^29^. It has also been demonstrated to be related to the amount of resources assigned to a particular activity or stimulus, meaning that short bursts of AE may aid in the allocation of attentional and memory resources, hence benefiting the executive control function^30^.

Results from our other findings indicate that cognitive processing speed and executive functions improved as a result of 8 weeks of AE training, as participants in the experimental group performed faster on both parts of TMT (TMTA and TMTB) after 8 weeks of AE intervention. This finding is consistent with the findings of many other studies^31^ where improvement with PA training has been seen in relation to both cognitive processing speed and executive functions^31^. However, one study demonstrated improvement in only TMTA and not in TMTB^32^, and another study showed no significant improvement in any of the TMT^33^. In addition, few studies showed improvement on either cognitive processing speed^34^ or on only executive functions^35^ following PA training. Furthermore, global cognition measured by the MMSE has been shown to improve with PA training in the previous studies^36^. In this study also, participants in the experimental group scored higher on the MMSE scale after 8 weeks of AE intervention. However, few studies report no significant improvement in MMSE scores following such training^31^.

Possible mechanisms for this improvement in cognitive functions with PA have been speculated, such as PA causes an increase in cerebral blood flow (CBF)^37^ and volume^38^ to meet the increased demands for glucose and oxygen in active neurons^39^, and PA can modify the brain structure by causing neuronal modification and creation of new neurons by various mechanism^40–42^. PA also stimulates the production of various important growth factors; these factors have a big impact on brain morphology^43,44^. Finally, PA has been linked to changes in the concentration of brain metabolites such as glutamate^45^. Remarkably, all of the above-discussed mechanisms could affect the EEG and ERP measures^46^, but why such physiologic changes would affect specific EEG and ERP bands is uncertain^47^. Given that such EEG alterations influence P300 component measures, the findings of this study can be interpreted as supporting the idea that greater circulatory capacity causes ERP changes.

This study also examined the association between neurophysiological and neuropsychological measures. The main findings of this study are as follows.

- P300 latencies are not associated with TMT, MMSE, and ECog. These results are in line with previous studies, which revealed no significant association between P300 Lat and TMT^48^, and between P300 Lat and MMSE^49^. However, there are few contradictions to our studies, which demonstrate a correlation between P300 Lat and TMT, and between P300 Lat and MMSE^50^. These contradictions may be explained by the use of a different sample size and different study populations in the present study as compared to the previous study^50^. Furthermore, the P300 correlate may be more sensitive to individual differences than other neuropsychological measures, which is another explanation for the lack of a meaningful association^51^.
- P300 amplitude is not associated with TMT, MMSE, and ECog. These findings are supported by previous studies that revealed no significant association between P300 Amp and TMT in schizophrenic patients^52^, and between P300 Amp and MMSE^53^. Our study, on the other hand, demonstrated a correlation between P300 Amp and TMT in obsessive compulsive disorder (OCD) patients^52^, and between P300 Amp and MMSE^50^.

Considering all the facts and findings, this study showed that 8 weeks of AE training may be a promising approach to improve cognitive functions in depressed older adults with MCI. However, this study had some limitations. Despite the claimed underlying mechanism in animal subjects, the evidence for ERP-P300 in humans is less persuasive, possibly because biological variables other than exercise may be at work^3^. Furthermore, considering relatively small number of patients, the question arises about the effectiveness of acute AE training. Furthermore, being conscious of one's goal to exercise can cause CNS arousal, which is an inevitable constraint of our study. We also did not investigate the long-term benefits of AE training. These are critical challenges that should be addressed in future research.
